# GNE myopathy: from clinics and genetics to pathology and research strategies

**DOI:** 10.1186/s13023-018-0802-x

**Published:** 2018-05-02

**Authors:** Oksana Pogoryelova, José Andrés González Coraspe, Nikoletta Nikolenko, Hanns Lochmüller, Andreas Roos

**Affiliations:** 1 Institute of Genetic Medicine, International Centre for Life, Central Parkway, Newcastle upon Tyne, UK; 2 Leibniz-Institut für Analytische Wissenschaften - ISAS - e.V, Biomedical Research Department, Otto-Hahn-Str. 6b, 44227 Dortmund, Germany; 3 Present Address: Department of Neuropediatrics and Muscle Disorders, Faculty of Medicine, Medical Center – University of Freiburg, Freiburg, Germany; 4 Centro Nacional de Análisis Genómico, Center for Genomic Regulation (CNAG-CRG), Barcelona Institute of Science and Technology (BIST), Barcelona, Catalonia Spain

**Keywords:** GNE myopathy, Distal myopathy, Sialic acid, Nonaka disease, HIBM, QSM, DMRV

## Abstract

GNE myopathy is an ultra-rare autosomal recessive disease, which starts as a distal muscle weakness and ultimately leads to a wheelchair bound state. Molecular research and animal modelling significantly moved forward understanding of GNE myopathy mechanisms and suggested therapeutic interventions to alleviate the symptoms. Multiple therapeutic attempts are being made to supplement sialic acid depleted in GNE myopathy muscle cells. Translational research field provided valuable knowledge through natural history studies, patient registries and clinical trial, which significantly contributed to bringing forward an era of GNE myopathy treatment. In this review, we are summarising current GNE myopathy, scientific trends and open questions, which would be of significant interest for a wide neuromuscular diseases community.

## Background

### History of GNE myopathy

GNE (bifunctional UDP-N-acetylglucosamine 2-epimerase/N-acetylmannosamine kinase) myopathy has been described for first time in 1981 by Ikuya Nonaka and colleagues as a distal myopathy with rimmed vacuoles and lamellar (myeloid) body depositions thus receiving the name of “Nonaka Distal Myopathy” or “Distal Myopathy with Rimmed Vacuoles” (DMRV) [1]. In 1984, Argov Zohar described a unique disorder presenting in 4 Iranian-Jewish families as a “Rimmed Vacuole Myopathy” or “Quadriceps Sparing Myopathy” (QSM) with characteristic clinical features [2]. Later, this disorder was also termed as “Hereditary Inclusion Body Myopathy” (HIBM) or hIBM, due to the histological similarities to Inclusion Body Myositis (IBM) [3].

In 1995, Mitrani-Rosenbaum and co-workers linked the origins of Persian Jewish QSM to chromosome 9 [4]. Approximately two decades after, in 2001, the Mitrani-Rosenbaum group identified mutations in the causative gene *GNE*, which encodes for the N-acetylglucosamine epimerase/ N-acetylmannosamine kinase (GNE) [5, 6]. The identified gene confirmed that those myopathies (DMRV, QSM, HIBM and IBM2) in fact represent the same neuropathological condition [6]. However, since the identification of *GNE* as the disease causative gene, the different historic names for this disorder continue to be used by research groups worldwide. For that reason, a consortium of researchers working on various aspects of this disease has decided in 2014 to unify the name and call it GNE myopathy [7].

## Clinical presentation

### Symptoms

**Fig. 1:**
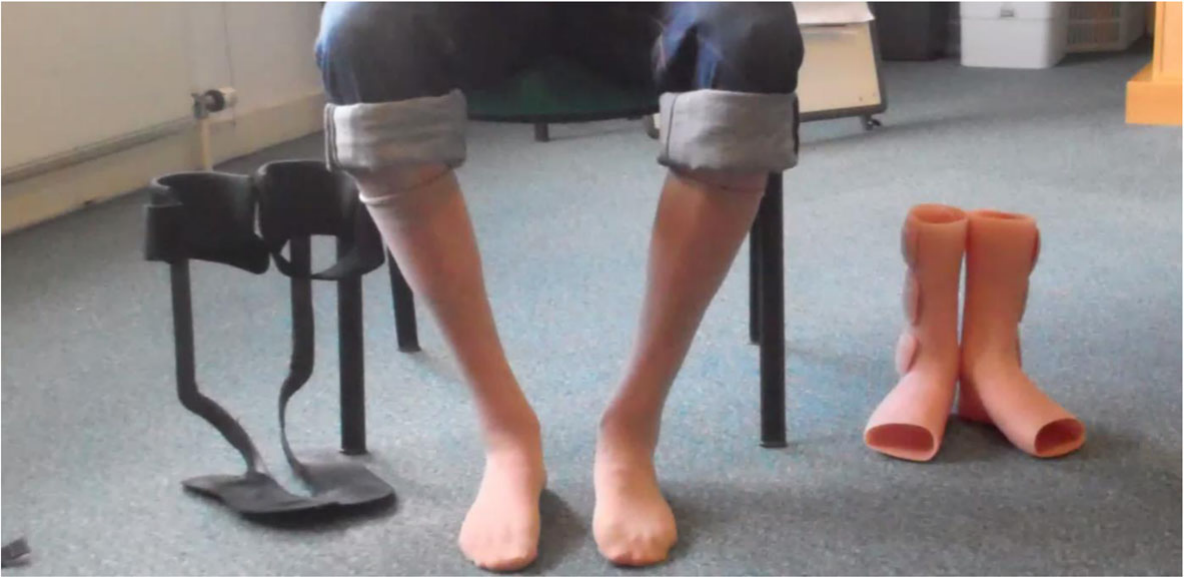
Distal muscle weakness in a GNE myopathy patient

GNE myopathy has an estimated worldwide prevalence of 1/ 1.000.000 [8, 9]. The spectrum of classical clinical presentations which was initially described in 1981 and 1984 remained unchanged [1, 2] and was complemented with broader spectrum of rarer and cohort specific symptoms. The first appearance of symptoms occurs most frequently in the third decade of life, although, few early onset cases (at 10 years of age) and late onset in the 5th decade have been reported [3]. The typical clinical presentation begins with distal weakness in the legs (foot drop) due to distal leg muscle weakness (Fig. 1), followed by slowly progressing muscle weakness and atrophy of lower (more frequent on the tibialis anterior [10]) and upper extremity muscles with relative sparing of the quadriceps [2]. Notably, the presentation of strong quadriceps in spite of major involvement in other leg muscles is still the best clinical signpost for the diagnosis of GNE myopathy as it is rarely found in other neuromuscular disorders [3].

The cause of the quadriceps sparing remains one of the enigmas of this condition [11]. The discovery of molecular mechanisms explaining the observation of a prevented muscle group might open new avenues for the development of further therapeutic intervention concepts [3]. Slow progression to the proximal musculature and the upper limbs warrant that patients may maintain independent walking for long time relying on the hip constitution [12]. Majority of the GNE patients retain the quadriceps sparing through several decades, while a minority (5%) have various degrees of quadriceps weakness early on [3].

A pattern of muscle weakness in upper limbs is variable and can mimic scapuloperoneal syndrome or include various degrees of hand weakness [8]. It has been described that patients with onset in proximal leg muscles might mimic an unusual pattern of limb girdle muscular dystrophy [13]. Consequently, this unusual clinical presentation may delay diagnosis but in retrospect both clinical and imaging features show that the posterior thigh muscles become markedly affected while the quadriceps is spared [8]. A recent study reported that “Beevor sign” is a common feature in GNE patients of Indian origin. “Beevor sign” is an upward movement of the umbilicus on neck flexion, indicating weakness of hip flexors and lower abdominal muscles compatible with an ascending pattern of muscle involvement [9]. This sign is characteristic of T9-T10 spinal cord injury and fascioscapulohumeral muscular dystrophy (FSHD) and observation of it in GNE myopathy has so far been cohort specific.

Other than for the muscle weakness and atrophies, the neurological examination is usually unremarkable without sensory disturbances, normal or low (due to muscle weakness) tendon reflexes, and normal cranial nerves examination. GNE myopathy is not associated with cognitive impairment.

### Muscle imaging

Imaging of skeletal muscles, especially MRI, is becoming more available in clinical practice and serves as a valuable non-invasive tool helping to better diagnose patients at early stages. Both T1 and T2-weighted sequences are used to get a comprehensive picture of the nature of muscle damage. Canonical presentation of GNE myopathy often reflects clinical presentation, where distal leg muscles, specifically anterior compartment, are severely affected at early stages of the disease, while quadriceps stays preserved over a long period of time, especially compared to the posterior compartment thigh muscles significantly replaced by fatty tissue. Selective quadriceps sparing, is often symmetrical, but noticeable degree of asymmetry has also been reported [14].

A retrospective systematic review of 13 GNE myopathy patients describes detailed assessment of 37 muscles at different stages of the disease [15]. The authors find that the following muscles were consistently involved at early stages in patients with typical and atypical clinical presentation: biceps femoris short head, gluteus minimus, tibialis anterior, extensor hallucis and digitorum longus, soleus and gastrocnemius medialis. They also observed a more selective sparing of quadriceps with vastus lateralis been the least affected part, even at advance stages of the disease, while rectus femoris, vastus intermedius and vastus medialis showed variable degree of fatty replacement.

Pelvic muscles and muscles constituting abdominal wall are not affected at early stages of the disease, but fatty and fatty-fibrous tissue infiltration of those muscles appear with the disease progressing further [16]. This may lead to difficulty keeping balance, flabby abdomen and positive Beevor’s sign, where proximal supra-umbilical part of rectus abdominis is replaced by fatty tissue, while infra-umbilical part is preserved [9]. Pelvic muscle MRI also shows abnormal iliopsoas, pectineus and gluteus minimus [9], gluteus maximus [17], medius [16].

For differential diagnosis, it is important that simultaneous involvement of semimembranosus, semitendinosus and tibialis anterior point towards GNE myopathy and helping to distinguish from other myopathies [16].

Muscles in younger patients that appear normal in T1, occasionally show hyperintensities in T2-weighted sequences, which may indicate a degree of inflammation [16]. This corresponds to biopsy findings, where signs of inflammation were found in some GNE patients at early stages of the disease [18].

### Neurophysiology

Needle electromyography (EMG) shows myopathic changes in the examined muscles [16, 17, 19]. Spontaneous activity in form of fibrillation potentials and positive sharp waves might be detected [20]. The EMG findings usually correlate with the clinical presentation. Thus, since anterior compartment of the lower limb gets affected in the first instance, EMG myopathic features are also more evident when assessed in this compartment [17]. Occasionally, EMG findings in GNE myopathy can be difficult to interpret and be reminiscent of EMG patterns in active myositis. This may have been the case in a patient, where GNE myopathy (onset at age 42) was preceded by systemic lupus erythematosus (positive antinuclear antibodies) and arthritis (onset at age 23) [21], but there are also some cases of GNE myopathy reported that showed strong inflammatory infiltrates histologically [18].

### Lung function test

It is generally considered that GNE myopathy does not predispose to respiratory failure. Relatively large cohort studies in the UK and Iran reported that respiratory function was not affected and FVC was normal in all patients [14, 20]. A more systematic prospective study, followed 24 patients for 1 year in Japan, showed that respiratory function is preserved in ambulant GNE patients and there were no changes in FVC over a year. In non-ambulant subset of the patients (*n* = 15) mild to moderate decrease of FVC (mean 74.5% SD ± 19.3%) was observed and it dropped further over the observation year (mean 69.8% SD ± 19.2, *p* = 0.034). A small number of severely affected patients have been reported using nocturnal non-invasive positive pressure ventilation (NPPV) [22]. Mild to moderate decrease of FVC (60-75%) was also observed in another cohort study, noting that respiratory muscles were only sub-clinically affected even at advance stages of the disease in bedridden patients [10].

These findings suggest no additional risk of respiratory failure to ambulant patients; non-ambulant patients might be at higher risk and therefore annual monitoring of respiratory function in non-ambulant GNE patients may be advisable to timely manage the situation in case of significant decrement of lung function.

### Cardiac studies

There is a limited number of studies, which systematically assessed cardiac function in GNE patients. Based on the limited number of studies, shared clinical experience of specialised neuromuscular centres and case reports, it considered that cardiac impairment is not linked to GNE myopathy. Here we will refer to two large studies, which specifically addressed analysis of cardiac function:

The first study assessed cardiac function in 33 Roma patients, using ECG and EchoCG. Minor to mild structural and rhythm abnormalities were detected in nearly half of the evaluated patients, such as impaired relaxation and repolarization. Three patients had borderline ejection fraction (EF – 50–55%) values. For the data interpretation, it is important to note that some of the above findings are subclinical, and patients had other co-morbidities and cardiovascular risk factors e.g. smoking (in all patients), hypertension and/or diabetes (in 18%).

A prospective natural history study conducted in Japan, followed 24 patients for 1 year and conducted ECG, Holter ECG and UCG. Two of these patients presented with minor to moderate conduction and rhythm abnormalities i.e. right bundle branch blocks (one complete and one incomplete), a 1st degree atrioventricular block with sinus bradycardia due to beta-blocker use, and a non-specific ST-T change (but normal UCG), sinus tachycardia and non-specific ST-T changes. Ejection fraction was normal in all patients. Patients with ST-T changes had diabetes mellitus and/or hypertension. The study did not show any disease-related abnormalities or any increased risk of cardiomyopathy in ambulant or non-ambulant GNE patients [22].

Presented data did not show any disease specific abnormalities or a consistent link between the GNE myopathy and increase the risk of conduction, arrhythmia, structural or functional cardiac defects.

### Blood tests

Blood tests, routinely available in a clinic, indirectly reflect muscle damage, i.e. mild to moderate CK elevation [14], sometimes with a mild ALT elevation (GGT normal) and low or normal creatinine. In non-ambulant patients CK could be within normal range or lower, in accordance with the reduced muscle bulk. A summary of main information related to GNE myopathy and its clinical presentation is given in Table 1.

**Table 1:** GNE myopathy at a glance

*GNE myopathy at a glance*	
Onset	20s
Prevalence	1/ 1.000.000
Genetic	Autosomal-recessive
First symptom	Foot drop
Additional symptoms	Hand weakness
Unusual mimicking of other myopathic diseases	Scapuloperoneal syndrome, LGMD, CMT, MTM, LGMD2B
Quadriceps sparing	Good strength in 95% of the patients
Progression	Slowly from distal to proximal
Beevor’s sign	Positive in 90% of an Indian cohort
Cardiac involvement	Risk of cardiomyopathy is not increased
Respiratory involvement	Mild to moderate decrease in FVC in non-ambulatory patients

An anecdotal case of mild to moderate thrombocytopenia have been reported in two siblings with GNE myopathy. Platelet levels varied between 1.1 × 10^9 /L and 16.2 × 10^9 /L. Thrombocytopenia, was characterized by shortened platelet lifetime rather than ineffective thrombopoiesis, has been observed since infancy. Genetic causes of persistent thrombocytopenia were excluded, and it was suggested that low platelet count maybe linked to GNE myopathy [23].

## Genetics

The *GNE* gene is located on chromosome 9 and consists of 13 exons. Each of the individual GNE mRNA splice variants consist of fewer exons and are two major isoforms are existing: hGNE1 (GenBank NP_005467) – a major muscle transcript, and hGNE2 isoform (NP_001121699) – the longest known sequence to up till now. hGNE1 was originally described as the GNE protein which covers 722 amino acids and is, confusingly, encoded in GenBank by mRNA transcript variant 2 (NM_005476). hGNE2 isoform covers 753 amino acids and is encoded by the longest GNE mRNA transcript, variant 1 (NM_001128227) [7]. In the scientific reports, case reports and cohort studies, mutations are most commonly reported according to hGNE1 or hGNE2 nomenclature. Notably, hGNE2 differs from hGNE1 by 31 amino acids or 93 base pairs. Thus, a particular mutation nomenclature can easily be converted in accordance to the preferred sequence.

Spectrum of disease causing mutations is wide and constantly growing. Currently, over 150 mutations are known to be causative for GNE myopathy [24]. Most of these mutations are sporadic or seen in several families or single cases only. Several mutations have been identified as founder or recurrent mutations [6, 10, 14, 25, 26]. These mutations are observed in relatively high frequency in Japan, Middle East, Roma population in Bulgaria, China and UK (Table 2.). Most of the currently known pathogenic variants are missense mutations; other mutations such as insertions, deletions, large deletions, intronic mutations [27], and splice site mutations [20, 28] have also been identified, but are far less common. Rare cases, clinically manifesting as GNE myopathy, but in absence of two recessive mutations could create a difficulty for molecular diagnosis. These clinically diagnosed GNE-cases could be caused by o a more complex molecular genetic rearrangement, such as copy number variation, large deletions [29], or deletions leading to Alu-mediated recombination [30]. Remarkably, so far, no patient has been identified carrying two nonsense or frameshifting mutations, suggesting that some basic activity of GNE is required during early development. Surprisingly, asymptomatic cases with confirmed two disease causative mutations have been described in the literature. This observation could indicate an incomplete penetrance of the disease, or even the significance of other (rescue) factors that can mitigate the symptoms.

**Table 2:** List of most commonly identified GNE mutations by the geographical region

Geographic region or country	Founder or high frequency mutation (hGNE2)
United Kingdom	Exon 8 p.Ala662Val Exon 12p.Asp409Tyr
Bulgaria (Roma population)	Exon 11 p.Ile618Thr
Middle East	Exon 7 p.Met743Thr
India	Exon 12 p.Val727Met
Japan	Exon 3 p.Asp207Val Exon 10 p.Val603Leu

## Genotype-phenotype correlation

A potential link between a genotype and a corresponding phenotype has been studied in vitro on cell and enzyme levels as well as based on patient cohort findings. In the context of in vitro studies, it is important to note an effect of various *GNE* mutations on the enzymatic activity of the resulting mutant proteins has been suggested: *E. coli* and insect cell models showed that indeed epimerase and kinase enzymatic activity significantly varied among selected mutations [31]. Primary muscle cells with *GNE* mutations confirmed a significant reduction in sialic acid levels [32].

Cohort based studies showed marked variability in severity of the disease [33] suggesting that certain point mutations are linked to age at onset, presenting symptoms, severity and speed of the disease progression [20, 26]. The largest cohort based study suggests that phenotypical differences between homozygous and compound heterozygous carriers; in this case one of the most common mutations in Japan p.Asp207Val seems to predispose to later onset and milder phenotype as opposed to p.Val603Leu [28]. However, phenotypical studies in patients homozygous for a single mutation demonstrates significant inter- and intra- familial variability [10] suggesting that the type of the *GNE* mutation only partially contributes to the individual variability and severity of the disease. Of course, the common problem of ultra-rare disease studies into consideration, all *GNE*-population based studies were significantly underpowered from statistical point of view. Hence, a very reliable link between genotype and phenotype is still lacking and must be deciphered.

## Biopsy findings and pathophysiological studies on patient-derived muscle

According to the current literature, majority of muscle biopsies derived from GNE myopathy patients are pathologically characterized by the presence of small angular fibres, formation of rimmed vacuoles and deposition of various proteins in the muscle fibres [34]. Further pathological hallmarks include the presence of intracellular Congo red-positive depositions in vacuolated or non-vacuolated fibres. The rimmed vacuoles can especially be found in atrophic fibres, which also occasionally contain the congophilic material immune-reactive to beta-amyloid, lysosomal proteins, ubiquitin and tau proteins. Inflammatory cell infiltration can also occasionally be found suggesting that muscle inflammation is not sufficient to exclude the diagnosis of hereditary inclusion body myopathy/ GNE myopathy [18]. The presence of inflammatory processes has been observed at early stages of the disease suggesting that the time point of biopsy procedure has a significant influence on pathological findings. Of note, based on the distal nature of this myopathic disease, those neuropathological findings refer to the distal muscles such as gastrocnemius muscle. However, other authors also refer to affection of proximal muscles such as biceps bracchii and the quadriceps muscle.

In muscles of GNE myopathy patients, immunohistochemistry allowed the identification of the GNE protein in sarcoplasm and specifically in myonuclei as well as within rimmed vacuoles. These vacuoles were also immunoreactive for nuclear proteins. In addition, measurements of the size of myonuclei in muscle biopsy specimen from GNE patients compared to those derived from ALS patients revealed a significantly larger mean size in muscle fibres of GNE patients than in ALS patients. The combined data suggest that myonuclei are involved in the formation of rimmed vacuoles in GNE myopathy and that mutant GNE in myonuclei seems to play some role in this process [35]. In contrast to this study, results of Krause and co-workers indicate that the GNE protein is expressed at equal levels in muscle fibres of patients and normal control subjects and that the GNE protein did “solely” mislocalization in skeletal muscle of patients. Therefore, the authors concluded that impaired GNE function, rather than expression or mislocalization, may be the key pathogenic factor in the disease. Moreover, they postulate that for diagnostic purposes, direct GNE gene testing will remain the mainstay and is not aided by immunohistochemistry or immunoblotting using antibodies against the GNE protein [36]. The lack of a GNE-antibody suitable for diagnostic management of GNE-patients moreover supports this proposed diagnostic procedure.

Already in 2004, biochemical analysis demonstrated decreased reactivity of skeletal muscle glycoproteins with the lectins recognizing sialic acid residues, suggesting that hyposialylation of glycoproteins may be involved in the etiology of GNE myopathy [37]. In addition, a study performed by Voermans and colleagues [38] also indicated reduced sialylation of glycoconjugates based on PNA lectin staining in GNE patient muscle sections compared to control muscle. Leoyklang and co-workers analysed the sialylation status of plasma and skeletal muscle proteins in a biomarker study. Muscle biopsy specimen derived from GNE patients showed hyposialylation of predominantly O-linked glycans thus suggesting that perturbed protein function based on impaired post-translational protein-modification is part of the etiology of GNE myopathy [39]. Huizing and co-workers studied the glycosylation status of alpha-dystroglycan in muscle biopsy specimen derived from GNE myopathy patients of non-Iranian Jewish origin. Remarkably, in all four muscle biopsies almost absent or markedly reduced immunolabeling with two different antibodies (VIA4 and IIH6) to glycosylated epitopes of alpha-dystroglycan could be observed. In this context, it is important to note that normal labelling was found using antibodies to the core alpha-dystroglycan protein, beta-dystroglycan, and laminin alpha-2. This finding suggested that GNE myopathy may fall under the category of a so-called “dystroglycanopathy” [40]. However, in another study, Broccolini and co-workers [41] also investigated alpha-dystroglycan (alpha-DG) immunoreactivity in 5 GNE myopathy patients. Their immunocytochemical and immunoblot studies revealed that alpha-DG extracted from muscle biopsies derived from GNE myopathy patients was normally expressed and displayed its typical molecular mass. However, further immunoblot analysis on the wheat germ lectin-enriched glycoprotein fraction of muscles and primary myotubes showed a reduced amount of alpha-DG in 4 out of 5 GNE myopathy patients (compared to control muscles). As the altered lectin-binding behavior (reflecting a partial hyposialylation of alpha-DG) did not affect the laminin binding properties of alpha-DG, the authors concluded that subtle changes within the alpha-DG glycosylation pattern most likely do not play a key pathogenic role in GNE myopathy [41]. Further studies such as glycoproteomics may be needed utilizing a higher number of patient and control samples to draw a final conclusion.

Proteomic profiling (two-dimensional gel electrophoresis (2-DE) and iTRAQ) has been performed on muscle cultures and biopsies of GNE myopathy patients. Out of the 400 proteins identified in biopsies by iTRAQ, 41 showed altered expression while the 2-DE analysis on biopsies revealed 26 differentially expressed proteins. Notwithstanding the fact that two different sources for protein extraction (muscle primary cultures versus muscle biopsies) have been utilised and that two different methods have been applied, the proteins identified with altered abundances in each of the analyses were mainly involved in the same pathways, ubiquitination, stress response and mitochondrial processes. Interestingly, the most robust cluster was assigned to cytoskeleton and sarcomere organisation. Thus, these findings indicate a possible function of GNE in the muscle filamentous apparatus that could be involved in the pathogenesis of the myopathy [42]. In another study to elucidate the pathological mechanisms leading from the mutated GNE to the myopathic phenotype, Eisenberg and colleagues [43] attempted to identify early manifesting downstream events. For that purpose, the genomic expression patterns of muscle specimens from 10 GNE myopathy patients carrying the p.M712 T mutation and presenting mild histological changes were compared with 10 healthy matched control muscles using GeneChip expression microarrays. Three hundred seventy four differentially expressed genes were identified. Approximately 20% of the overall differentially expressed mRNAs of known function were found to encode for proteins implicated in various mitochondrial processes, revealing mitochondrial pathway dysregulation. This finding is in agreement with the results of the proteomic studies. Further morphological analysis by confocal microscopy showed a high degree of mitochondrial branching in cells of GNE myopathy patients. The involvement of mitochondrial processes in the pathophysiology of GNE myopathy reveals an unexpected facet, which could at least partially explain the slow evolution of this disorder [43].

ER-stress and the activation of the unfolded proteins response (UPR), as the respective cellular defence mechanism, have been described in sporadic inclusion-body myositis (sIBM), In contrast, UPR key players (ATF4, ATF6, BiP and XBP1) in muscle of GNE patients lacked any evidence of UPR induction. However, cultured GNE-h-IBM muscle fibers had a robust UPR response to experimental ER stress stimuli, suggesting that the GNE mutation per se is not responsible for the lack of UPR in GNE-h-IBM biopsied muscle [44]. In contrast, activation of the unfolded protein response as well as the ubiquitin proteasome system along with autophagy have been described in muscle biopsy specimen of eight patients with GNE myopathy by another group [45] using immunofluorescence and immunoblotting. Elevated protein abundance of BiP/GRP78, GRP94, calreticulin and calnexin (all are major chaperones controlled be the unfolded protein response) was demonstrated. Moreover, VCP (important for the ER-associated degradation pathway) was increased. Increased proteasome activities were shown by forced cleavage of fluorogenic substrates. 20S proteasome subunits, three main proteasome proteolytic activities, and the factors linking UPS and autophagy system were also increased. The authors concluded that activation of these cellular defence mechanisms resulted from intracellular beta amyloid (Aβ) accumulation. Of note, Fischer and colleagues [46] revealed that the mRNA-expression of APP significantly correlated with the expression of αB-crystallin (a molecular chaperone) and several pro-inflammatory and cell-stress-associated markers as NCAM, IL-1β, TGF-β, CCL-3, and CCL-4. Normal appearing fibres displayed an overexpression of these molecules, and their elevated expression is compatible with an activation of cellular defence mechanisms.

## Disease models and pathomechanisms

**Fig. 2:**
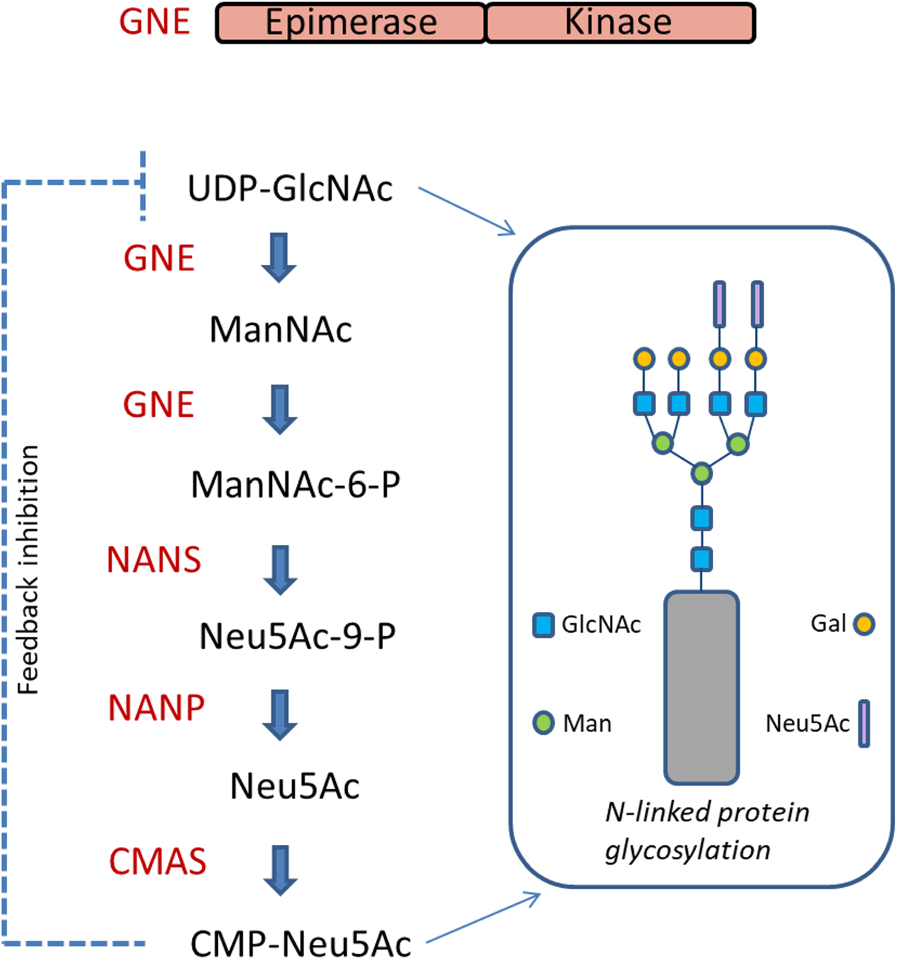
The bifunctional enzyme UDP-GlcNAc 2-epimerase/ ManNAc kinase (GNE/MNK), encoded by the GNE gene, catalyzes the first two committed, rate-limiting steps in the biosynthesis of N-acetylneuraminic acid (sialic acid)

Sialic acid is an acidic monosaccharide that modifies non-reducing terminal carbohydrate chains on glycoproteins and glycolipids and plays an important role in different processes such as cell-adhesion and cellular interactions. Sialic acid has been implicated in health and disease and is found in terminal sugar chains of proteins modulating their cellular functions. As UDP-N-acetylglucosamine 2-epimerase/N-acetylmannosamine kinase (GNE) is the key enzyme for the biosynthesis of sialic acid (Fig. 2) it is without any doubt that perturbed function of the protein results in biochemical consequences. Indeed, GNE mutations can result in two human disorders, GNE myopathy or sialuria. Moreover, it has been demonstrated that GNE expression is induced when myofibers are damaged or regenerating, and that GNE plays a role in muscle regeneration [47]. However, correlation between mutation-associated reductions in sialic acid production and disease severity is imperfect and although the underlying pathophysiology is, at least partially, likely to result from perturbed post-translational protein modification (hyposialylation of glycoconjugates; Fig. 2), many mechanisms have been suggested as possible (downstream) cause of muscle degeneration such as defects in cytoskeletal network, sarcomere organization and initiation of apoptosis. Support for this hypothesis was presented by Salama and colleagues [48]. Myoblasts carrying a mutated *GNE* gene show a reduction in their epimerase activity, whereby only the cells carrying a homozygous epimerase mutation also present with a significant reduction in the overall membrane bound sialic acid. This finding indicates that although mutations in each of the two *GNE* domains result in an impaired enzymatic activity and the same myopathic phenotype, they do not equally affect the overall sialylation of muscle cells. Thus, the pathological mechanism of the disease may not exclusively be linked to the impaired sialic acid pathway [48].

### In vitro models

As it is known that the mutated hypofunctional GNE is associated with intracellular accumulation of amyloid β-peptide (Aβ) in patient muscles (see “biopsy findings and pathophysiological studies on patient-derived muscle” section) Bosch-Morató and colleagues [49] addressed the underlying mechanism by using C2C12 cells and demonstrated that systematic reduction of sialic acid favors Aβ1-42 endocytosis in a clathrin and heparan sulfate proteoglycan-dependent manner explaining the enhanced Aβ1-42 internalization in myoblasts from a GNE myopathy patient. As a consequence, reduced phosphor-AKT level accompanied by an increase of apoptosis marker proteins could be observed in the patient-derived cells.

To elucidate the role of GNE in cell apoptosis, Singh and Arya [50] used HEK293 cells overexpressing pathologically relevant *GNE* mutations. These disease-model cells display defective proliferation, reduced sialic acid-bound glycoconjugates level and increased apoptosis. Transmission electron microscopic studies revealed mitochondrial perturbations which is in line with altered mitochondrial transmembrane potential in cells lacking functional GNE. HEK293 cells in which *GNE* has either been knocked down or over-expressed with pathologically relevant *GNE* mutants (p.D207V and p.V603 L)show that mutant forms of GNE differ in their subcellular localizations from the wildtype protein and sialylation-studies of β1-integrin revealed hyposialylation along with mislocalisation to internal vesicles. This mislocalization could be restored upon supplementation with sialic acid. Fibronectin stimulation caused migration of hyposialylated β1-integrin to the cell membrane and co-localization with focal adhesion kinase (FAK) leading to increased focal adhesion formation. Hence, results of this study demonstrate that *GNE* mutations affect β1-integrin-mediated cell adhesion processes [51].

Patzel and co-workers [52] investigated the accumulation of glycosphingolipids by HPLC in patients’ and control fibroblasts and plasma. The mutant cells exhibited impaired GNE epimerase activity through a novel imino sugar resulting in an increase of both, neutral and sialylated glycosphingolipids. Interestingly, treatment of patient-derived fibroblasts with N-acetylmannosamine (sialic acid precursor downstream of GNE epimerase activity) ameliorated the increased glycosphingolipids concentrations. These data may lead to further studies of glycosphingolipids concentrations as a potential biomarker, not only for GNE myopathy, but also for other disorders of sialic acid metabolism. Indeed, when studying the tissue of *Gne* (p.M712 T/p.M712 T; according to the new nomenclature p.M743 T/p.M743 T) knock-in mice (described below in more detail) elevated glycosphingolipids concentrations could also be observed supporting the concept of glycosphingolipids concentrations as a biomarker [52]. By focusing on the same mutation in primary patient –derived myoblasts cultures, Amsili and colleagues [53] identified that although p.M712 T-mutant GNE and control myoblasts showed similar patterns of proliferation and differentiation, upon apoptosis induction, active forms of caspase-3 and -9 were strongly increased in p.M712 T-GNE cultures compared to controls, while pAKT, downregulated in controls, remained high in patient-derived cells. These results suggest impaired apoptotic signalling in GNE-mutant muscle cells. This observation accords with the findings of different scientific reports [49, 50]. As satellite cells enable muscle regeneration, these altered cellular processes most likely contribute to the muscle mass loss observed in patients [53].

Bennmann and co-workers [54] studied the molecular effect of a particular amino acid exchange (p.M743 T) found in severe cases of GNE myopathy and demonstrated that amino acid exchanges introducing potential phosphorylation/O-GlcNAcylation sites result in increased O-GlcNAcylation and increased stability of the mutant protein suggesting that the balance of phosphorylation and O-GlcNAcylation is involved in the modulation of efficiency GNE. In vitro investigation of the same mutation by surface plasmon resonance and microscale thermophoresis analysis revealed that wildtype GNE interacts with α-actinin 2 with a 10-fold higher affinity compared to the GNE-α-actinin 1 interaction (which has been described before; [55]). In contrast, p.M743 T GNE displays a 10-fold lower binding affinity to α-actinin 2. This pathophysiological finding is most likely based on perturbed protein-protein binding and leads to functional imbalance in skeletal muscle [56]. Further studies utilizing GNE p.M743 T-mutant myoblasts revealed increased level of activated PTEN and PDK1 [57].

In another study [58], BJAB K20 cells, as an in vitro system lacking endogenous GNE activity based on epigenetically-based silencing have been used to introduce expression of wild-type GNE or mutant forms. Latter ones either affected the kinase (p.M712 T) or the epimerase (p.D176V) domain. Moreover, an artificial open reading frame encoding a GNE protein lacking the epimerase domain has been introduced into BJAB K20 cells and subsequent. Lectin binding and mass spectrometry analysis revealed that GNE deficiency affects the structure of cell-surface glycans. Apart from low levels of sialylation, GNE-deficient cells produced distinct N-linked glycan structures with increased branching and extended poly-N-acetyllactosamine. Interestingly, N-linked glycans produced by GNE-deficient cells displayed enhanced binding to galectin-1, indicating that changes in GNE activity can alter affinity of cell-surface glycoproteins for the galectin lattice. This in turn indicates a pathomechanism by which GNE activity might affect signaling through cell-surface receptors [58].

Grover and co-workers used Dictyostelium discoideum (species of soil-living amoeba) to study expression and secretion of wildtype and mutant forms of GNE. Whereas upon starvation, wildtype GNE (as a functionally fully active enzyme) was secreted into the medium from secretory vesicles, secretion of both epimerase and kinase mutant forms of GNE was found to be drastically reduced. This alternative in vitro system can be used for biophysical characterization of GNE and may give an indication of the pathogenicity of mutant variants of the protein to evaluate the potential pathogenicity of newly identified GNE mutations [59].

### Mouse models

Malicdan and colleagues generated the *Gne*^−/−^*GNE*D176V-Tg mouse model which exhibits late onset progressive muscle weakness and atrophy and pathological changes like those observed in patients. Those changes included presence of rimmed vacuoles, especially in atrophic fibers, which also occasionally contain congophilic material. In addition to these myopathic findings, the mouse model also showed hyposialylation of serum and other tissues from birth and exhibited late onset myopathy accompanied by mild serum creatine kinase elevation from 21 weeks of age [60]. Moreover, it has been demonstrated that the myopathic phenotype was prevented by oral administration of N-acetylneuraminic acid, N-acetylmannosamine, and sialyllactose (favorable improvement in survival rate, motor performance, muscle force, muscle atrophy, and muscle degeneration), suggesting that hyposialylation is an important factor in the pathogenesis of GNE myopathy [61]. Several synthetic sugar compounds that may significantly increase sialylation and show measurable effects were screened with the result that tetra-O-acetylated N-acetylmannosamine increased cell sialylation most efficiently, leading to a more dramatic, measurable effect and improvement in muscle phenotype [62]. These findings provided a proof of concept in sialic acid-related molecular therapy with synthetic monosaccharides. In another follow-up study, temporal changes in the overall motor performance of this model were addressed: studies revealed muscle weakness, decreased whole muscle mass and cross-sectional area (CSA), and reduced contractile power in an age-related manner. Investigation of single-fiber CSA supported the finding of muscle atrophy and displayed affection of both, type I and type II fibers. In older animals, RVs and intracellular inclusions were seen in type IIA fibers, further aggravating reduction of force and specific increase in twitch-tetanus ratio. This effect was – in accordance with the nature of a distal myopathy in patients – very pronounced in gastrocnemius muscles. These results implicate the important role of atrophy in the pathophysiology of GNE myopathy [63]. Yonekawa and co-workers [64] examined the efficacy of sialic acid supplementation on symptomatic Gne^−/−^GNED176V-Tg mice presenting with active progressive muscle degeneration. Hereby, the therapeutic effect of a less metabolized sialic acid compound (6′-sialyllactose) or free sialic acid (N-acetylneuraminic acid) was studied by oral, continuous administration to 50-week-old mice for 30 weeks. As readout-measures, motor performance in living mice and spontaneous locomotion activity on a running wheel have been investigated at 50, 65, 72 and 80 weeks of age. In addition, fibre size, force production and general pathology were studied in gastrocnemius muscle along with level of sialic acid. Notably, spontaneous locomotion activity was recovered in 6′-sialyllactose-treated mice, while NeuAc-treated mice slowed the disease progression and 6′-sialyllactose-treatment showed a positive effect towards restoration of hyposialylation in muscle and consequently to robust improvement in the muscle size, contractile parameters, and pathology. This beneficial effect could not be observed for NeuAc. Hence, the results indicate that GNE myopathy can be treated even at a progressive stage and 6′-sialyllactose has more remarkable advantage than free sialic acid, providing a conceptual proof for clinical use in patients.

A transgenic mouse that expressed the human GNE p.V572 L mutation (the most prevalent among Japanese GNE patients) has been generated, and crossed this with Gne(+/−) mouse to obtain *Gne*^−/−^hGNEV572L-Tg animals. Mutant mice exhibit marked hyposialylation in serum, muscle and other organs such as kidney. Notably, reduction in motor performance can only be seen from 30 weeks of age and a compelling finding is the development of beta-amyloid deposition in myofibers by 32 weeks. Latter one clearly precedes rimmed vacuole formation at 42 weeks [65]. Interestingly, the Gne^*−/−*^hGNEV572L-Tg animals moreover exhibit hyposialylation and intracellular amyloid deposition before the characteristic rimmed vacuoles can be detected, suggesting that autophagy might be a downstream effect to hyposialylation and amyloid deposition in GNE myopathy [66]. In 2012, Ito and colleagues [67] further reported that renal pathology was based on hyposialylation of podocalyxin. Administering Neu5Ac to the mutant mice from embryonic stages significantly suppressed the renal pathology and partially recovered the glomerular glycoprotein sialylation. However, activation of the unfolded protein response in kidney and skeletal muscle has not been studied in this mouse model.

Cho and co-workers [68] performed a study utilizing another in vivo model of GNE myopathy, the *Gne*^−/−^h*GNE*V207L-Tg mice [60]. Results of their studies provided evidence for function of sialic acids as a ROS scavenger and thus improved the current understanding on how sialic acid deficiency contributes to disease pathology: their studies revealed that proteins decisive for proper muscle function and maintenance were highly modified by S-nitrosylation. Additionally, oxidative stress-responsive genes were significantly upregulated in hyposialylated murine muscles (same could be confirmed in patient derived muscle biopsies in turn highlighting the suitability of the mouse model) as a reaction to elevated production of reactive oxygen species (ROS). Notably, increasing overall sialylation by extrinsic sialic acid intake reduced ROS and protein S-nitrosylation. Notably, N-acetylcysteine (an antioxidant) intake ameliorated muscle weakness and atrophy in the mouse model [68]. Moreover, the authors provided molecular insights into the associated muscle fibre degeneration by demonstrating that two well-known muscle atrophy markers (atrogin-1/Fbxo32 and MuRF1/Trim63) present with elevated transcript level in the diseased murine muscle fibres, suggesting that common proteolytic systems of muscle atrophy are involved in the pathophysiology of GNE myopathy [68].

As the most frequent mutation in GNE myopathy patients is the Middle Eastern (Persian-Jewish) founder mutation p.M712 T, Sela and co-workers [69] generated Gne^(p.M712T/p.M712T)^ knock-in mice. Notably, a high mortality rate has been observed in the first generation based on renal failure. However, the following generations were classified into 3 phenotypic categories: severe, mild and without apparent phenotype. Further crossing of mice lacking an apparent phenotype allowed the establishment of a colony with long-term survival. These animals did not show any signs of a kidney phenotype but also no apparent muscle phenotype for up to 18 months of age. And although no clear correlation was found between the expression of the two *Gne* mRNA isoforms in skeletal muscle and genotype or phenotype, expression of isoform 2 mRNA was significantly higher in the kidney of Gne^(p.M712T/p.M712T)^ animals. Notably, expression of proteins involved in the modulation of the unfolded protein response such as BiP and CHOP as well as enhanced splicing of *Xbp1* has been found in the skeletal muscle but not in the kidney of the homozygous mutant animals. This observation supports the results of Li and co-workers [45] suggesting that activation of the unfolded protein response might prevent GNE-diseased muscle fibres from death (see below). Quantitative RT-PCR analysis of *St 3 gal5* (GM3 synthase) gene expression and HPLC-based quantification of GM3 ganglioside were conducted on Gne^(p.M712T/p.M712T)^ and control mice. Results showed that *St 3 gal5* mRNA levels were significantly decreased in skeletal muscle derived from the mutant animals. In accordance with this finding, GM3 ganglioside levels also showed a significant decrease in skeletal muscle derived from the mutant animals. Although Gne^(p.M712T/p.M712T)^ mice were described to suffer from severe glomerular proteinuria (see above), no GM3 alterations were noted in kidneys, suggesting a tissue specific alteration of gangliosides. Hence, the homozygous p.M712 T mutation in GNE hampers the muscle ability to synthesize normal levels of GM3 [70]. Another study focused on the beneficial effect of oral monosaccharide supplementation as a therapy to reverse renal and muscle hyposialylation. Both, effectiveness for prophylaxis (at the embryonic and neonatal stages) and therapy (after the onset of symptoms) were studied by evaluating renal and muscle hyposialylation: oral mannosamine (ManN), (but not sialic acid (Neu5Ac), mannose (Man), galactose (Gal), or glucosamine (GlcN)) administered to pregnant female mice has a prophylactic effect on renal hyposialylation, pathology and neonatal survival of mutant offspring, as already shown for N-acetylmannosamine (ManNAc) therapy [71]. As GNE myopathy patients require treatment in adulthood (after onset of symptoms), Niethamer and colleagues [71] additionally administered ManNAc (1 or 2 g/kg/day for 12 weeks), Neu5Ac (2 g/kg/day for 12 weeks), or ManN (2 g/kg/day for 6 weeks) in drinking water to 6 months old animals. Notably, all three therapies markedly improved the muscle and renal hyposialylation. This was clearly evidenced by lectin histochemistry for overall sialylation status and immunoblotting of specific sialoproteins. These combined findings clearly support further evaluation of oral ManNAc, Neu5Ac and ManN as a potential therapy for GNE myopathy.

In 2012, Mitrani-Rosenbaum and co-workers applied a gene therapeutic approach as an interventional concept to treat GNE myopathy: AAV8 viral vectors carrying wild type human GNE cDNA were able to transduce murine and human muscle cells carrying GNE mutations. Based on this promising finding, in the next step, the authors intravenously administered this viral vector to healthy mice allowing expression of the GNE mRNA (and of the co-expressed luciferase protein) for 6 months in skeletal muscles. Hereby, no pathological signs of focal or general toxicity, neither from the virus particles nor from the wild type human GNE overexpression could be observed. This sustained and safety expression of human GNE in normal mice after gene transfer based on AAV8 systemic delivery suggests that GNE-based gene therapy might represent a promising concept to treat the disease [72].

### Zebrafish model

By in situ hybridization and *Gne* promoter-driven fluorescent transgenic fish generation, Daya and colleagues [73] investigated the spatiotemporal expression pattern of the zebrafish gne gene and have shown that it is highly conserved compared with the human ortholog. Morpholino (MO)-modified antisense oligonucleotides-based gene depletion resulted in a significantly reduced locomotor activity accompanied by distorted muscle integrity, including a reduction in the number of muscle myofibres. Muscle fibre pathology was moreover confirmed by electron microscopy studies, where large gaps between sarcolemma could be detected. However, sarcomeric structures were maintained. The combined data highlight a prominent role of GNE also in zebrafish and suggest that the zebrafish model is a suitable animal model for further pathophysiological studies and/ or testing of therapeutic intervention concepts.

## Biomarkers

Valles-Ayoub and colleagues developed a method to allow detection of serum NCAM sialylation using Western blot, and tested serum samples from several GNE-patients. Their results showed a clear difference in polysialylated and hyposialylated forms of serum NCAM and revealed that NCAM is hyposialylated in patient serum samples, suggesting changes of NCAM-sialysation to a potential serum biomarker for GNE myopathy [74]. In this context, it is important to note that NCAM plays a crucial role for stability of (re)-innervated neuromuscular junctions [75] and that altered NCAM secretion might influence this event. However, systematic studies on neuromuscular junctions in GNE myopathy are still lacking. In addition, a systematic study of a large patient cohort (ideally with a diversity of *GNE* mutations) would be needed to define changes of NCAM-sialysation as a reliable serum biomarker for this disease.

Extent of pre-existing serum antibodies to rAAVrh74, rAAV1, rAAV2, rAAV6, rAAV8, and rAAV9 has been examined in patients suffering from Duchenne muscular dystrophy (DMD), Becker muscular dystrophy (BMD), inclusion body myositis (IBM), and GNE myopathy. The rationale behind this study was that recombinant adeno-associated virus (rAAV) is a commonly used gene therapy vector for the delivery of therapeutic transgenes in a variety of human diseases, but pre-existing serum antibodies to viral capsid proteins can greatly inhibit rAAV transduction of tissues [76]. Compared to serum samples derived from control individuals, patients with measurable titers to one rAAV serotype showed titers to all other serotypes analyzed. Hereby, average titers to rAAV2 showed to be highest in all patients. Of note, 50% of all IBM and GNE patients also had antibody titers to all rAAV serotypes, while only 18% of DMD and 0% of BMD patients did. These data indicate a concern of treatment blockage by pre-existing serum rAAV antibodies in GNE myopathy [76]. However, a systematic study of pre-existing serum rAAV antibodies in serum samples derived from animal models are still lacking and a potential positive result would not only further demonstrate their suitability as a good phenocopy of the human disease but also allow to systematically address the hypothesis that these pre-existing antibodies might negatively influence gene therapeutic concepts utilizing a viral approach.

As the pathophysiology of GNE myopathy presumably involves aberrant sialylation, sialylation status of blood-based glycans was studied as potential disease marker by Leoyklang and co-workers [39]. Compared to control samples, O-linked glycome of patients’ plasma showed increased amounts of de-sialylated Thomsen-Friedenreich (T)-antigen, and/or decreased amounts of its sialylated form (ST-antigen). Interestingly, hereby all GNE patients presented with increased T/ST ratios compared with controls. Further studies of muscle biopsy specimen derived from GNE patients showed hyposialylation of predominantly O-linked glycans. Based on their findings, the authors postulated that plasma T/ST ratios are a robust blood-based biomarker for GNE myopathy [39].

Myostatin is secreted primarily from skeletal muscle and can potently suppress muscle fiber growth and has hence the ability to regulate skeletal muscle mass. This in turn has sparked interest in the development of anti-myostatin therapies for a variety of muscular disorders. Burch and colleagues have measured the serum myostatin concentration in seven genetic neuromuscular disorder patient populations including GNE myopathy. For that purpose, immunoaffinity LC-MS/MS has been applied and average serum concentrations of myostatin in seven muscle disease patient groups, including GNE patients, were significantly less than in controls. Hereby, myostatin level correlated with clinical measures of disease progression in GNE myopathy. These findings suggest the potential of myostatin as a biomarker of disease progression in GNE myopathy [77]. However, further studies investigating larger patient cohorts would be helpful to determine the utility of myostatin as a reliable blood biomarker for GNE myopathy. In the same context, it would be very interesting to study the level of follistatin and correlate the myostatin/ follistatin ratios with the genotype as well as the severity of the disease. In addition, study of myostatin/ follistatin level in the above-mentioned mouse models would give further insights into their suitability as appropriate animals models for GNE-myopathy.

## Interventional strategies

### Therapy development

There is no approved treatment for GNE myopathy to date. Current patient management is focused on improving quality of life by addressing major symptoms. This includes physiotherapy, selection of walking assistive devices and orthoses, psychological support, pain management and nocturnal ventilation where relevant, mobility devices (e.g. wheelchair or scooter), carer help and alternative professional development.

Significant effort is currently being put in translational research to find a treatment for GNE myopathy. This includes clinical trial readiness and baseline data collection via national and international patient registries (Remudy “www.remudy.jp” and International GNE registry “www.gnem-dmp.com”). Multicentre Natural history studies conducted by NIH (USA) and Ultragenyx Pharmaceutical (USA) contribute to a systematic approach of studying versatile presentation of GNE myopathy and assessment of muscle decline in a given period of time.

Current statistics shows that Remudy registry has over 200 registered patients in Japan, GNEM-DMP has over 300 patients registered worldwide. Both registries are recruiting patients and collecting mandatory data items. In addition, International GNE registry collects medical history, longitudinal progression, and quality of life; Remudy collects clinical data on lung function, ambulation and CK level. Both registries communicate to registry participants to inform them about relevant scientific studies, advances in the research and patient advocacy meetings.

Two natural history studies of GNE myopathy are currently in progress. One is conducted by Ultragenyx Pharmaceutical (USA), this is multicentre, international study, with > 100 patients recruited and followed up between 1 and 4 years (www.clinicaltrials.gov ID NCT01784679). The study collects longitudinal data including medical history, serum biomarkers, physiotherapy and patient-self reported outcomes. Another study is a single-centre, prospective Natural History study conducted at the NIH (USA) that has recruited > 50 patients, with a plan to follow them up for up to 6 years (NCT01417533). The study evaluates muscle strength, function and subjective patient-reported outcomes, along with serum and urine biomarkers and muscle imaging. Preliminary results of the natural history studies, demonstrated in the neuromuscular meetings and conferences, show slow but measurable decline of muscle strength in upper and lower extremities and decline in general physical activity and ability to perform daily living activities over the time.

Disease pathomechanism and animal model studies suggested that supplementation of ManNAc or sialic acid is beneficial in GNE myopathy [61, 64]. Therefore, the idea of exogenous replenishment of Sialic acid was taken forward into clinical investigations. Several therapeutic approaches have been suggested as a potential treatment for GNE myopathy. Therapeutic approaches were based on the pathway of the disease, which affect Sialic acid synthesis, and effectively results in a sialic acid deficiency. The following compounds have been studied as a source of sialic acid supplementation: Aceneuraminic acid, ManNAc, and immunoglobulin (IVIG).

IVIG therapy was administered to 4 patients at a loading dose of 1 g/kg on two consecutive days followed by 3 doses of 400 mg/kg at weekly intervals. The study showed mild improvement in quadriceps, shoulder muscle strength and in 8 other muscle groups by the end of the study. Patients self-reported and objective measures were variable and not presented in detail. Immunohistochemical staining and immunoblotting of muscle biopsies for alpha-dystroglycan and NCAM did not show that IVIG treatment improves muscle syalilation. Although study showed some mildly positive signs and was tolerated without significant adverse events it was considered not to pursue IVIG therapy any further [78].

Aceneuraminic acid (Ace-ER) and ManNAc were studied to a much greater extent. Ace-ER went all the way from preclinical studies to the completing Phase 3 double blind placebo-controlled study in 2017. Preclinical studies and early clinical studies showed stabilisation and slower decline of muscle function [33, 79], (NCT02731690, NCT02736188, NCT01517880, NCT01830972, NCT01236898). Phase 2 clinical trial showed dose-dependent improvement in muscle strength relative to placebo in some muscle groups. Unfortunately, the Phase 3 study did not meet any primary or secondary end points and, therefore it was concluded that Ace-ER was safe and had no or very little effect on GNE myopathy progression (NCT02377921).

The intermediate of the sialic acid biosynthesis pathway - N-acetyl-D-mannosamine (ManNAc) is another potential therapeutic option. A Phase 1 trial (NCT01634750) of ManNAC is complete and a Phase 2 trial (NCT02346461) is currently ongoing. ManNAc is reported to be safe with recent publications suggesting that ManNAc restores the intracellular biosynthesis of sialic acid, including in patients homozygous for kinase domain mutations.

A single attempt of gene therapy in a GNE patient was documented in 2011 [78], the results showed modest improvement, but this therapy has never been followed up or tested in a well-designed and approved clinical trial.

Translational research projects, including clinical trial attempts presented here are in development and new results might appear soon. However, it is clear that any successful therapy development in this ultra-rare disease requires a substantial multicentre, international collaboration.

### Diet/ nutrition

Although patients suffering from GNE myopathy do not require any special diet, a benefit of consumption of food rich in sialic acid is very likely. Sialic Acid can be found in milk and dairy products (e.g. whey) as well as in some exotic meals like Chinese delicacy Yanwo. To date no research has been conducted to test the hypothesis that food supplementation of sialic acid has any benefit for the muscle strength in GNE myopathy. One observational study however suggests that consumption of traditional whey-based drink in Iran (called “Arshe” or “Lour”), rich in sialic acid may lead to a slight palliation of symptoms and perhaps to a delay in the age of onset. Although whey contains a high concentration of sialic acid, neither of these dietary substances has been scientifically evaluated for their specific components and their correlation with muscle strength or other objective measure. The authors of the paper discuss that in their opinion further analysis is required to systematically assess hypothesised effect of dietary supplementation of sialic acid.

### Physiotherapy

GNE myopathy is a slow progressing disorder which does not have a tendency to contractures. Physiotherapy and hydrotherapy are recommended under supervision of experienced physiotherapist. Unsupervised sessions can also be advised and planned by a specialist to be conducted at home by patient and their caregiver it has recently been shown that GNE myopathy patients may benefit from attending physical therapy or occupational therapy services to help maintain their functional capacity [12]. Taking the degree of disease progression in GNE myopathy into consideration, more research is required to define and improve training protocols and optimize exercise strategies and routine protocols for this condition. However, there is valid evidence supporting the safety of low to moderate intensity loading exercises [Error! Bookmark not defined.]. Additionally, exercise may enhance cardiovascular activity and may help to reduce experienced fatigue [80]. Inclusion of patients in decision making and planning of the exercise schedule and intensity will help to maximise outcomes and self-efficacy in people with this long-term condition [Error! Bookmark not defined.].

## Conclusions

GNE myopathy, discovered over 30 years ago, has now been studied on many levels ranging from cell and animal models, to systematic natural history and epidemiology studies in patients leading to the development of therapeutic strategies based on the substitution of sialic acid, which were tested in clinical trials. Classical presentation with juvenile or young-adult foot drop in the absence of sensory deficits, spared quadriceps and rimmed vacuoles in muscle biopsy specimens should lead to inclusion of GNE myopathy in differential diagnosis, followed by genetic testing. The disease management currently includes mobility assistance and support, physiotherapy and management of other associated symptoms (e.g. musculoskeletal pain, pressure sores), however no disease specific treatment is currently available. Ace-ER (sialic acid) was envisaged to be a first potential therapy for GNE myopathy and showed stabilisation of muscle function in phase 2 clinical trial, however a large and well-conducted double-blind, placebo-controlled phase 3 study did not support efficacy of this treatment regimen. Further research into the mechanisms underlying the disease, and into GNE enzyme function, maybe required to identify alternative treatment options. The GNE enzyme is involved in sialic acid production and forms a complex with α-actinin 2 which is important for the cell stability and contraction (https://www.ncbi.nlm.nih.gov/pubmed/27023225). In the same context, different potential biomarkers have been identified which might serve as potential readouts for future therapeutic interventions. Comprehensive studies using in vitro and in vivo models indicate activation of the unfolded protein response as an efficient protective mechanism in this disease. This may have therapeutic significance if the protective cascade could be activated by drug therapy.

## Abbreviations

2-DE: Reactive oxygen species+A20:B29
Ace-ER: Aceneuraminic acid
ALS: Amyotrophic lateral sclerosis
ALT: Alanine aminotransferase
APP: Amyloid precursor protein
BiP: Binding-immunoglobulin protein; also known as 78 kDa glucose-regulated protein (GRP78) or Heat shock protein family A member 5 (HSPA5)
BJAB K20 cells: Burkitt lymphoma cell line lacking UDP-GlcNAc 2-epimerase activity
CHOP: C/EBP-homologous protein
CK: Creatine kinase
CSA: Cross-sectional area
DMRV: Distal Myopathy with Rimmed Vacuoles
ECG: Electrocardiogram
EchoCG: Echocardiography
EF: Ejection fraction
EMG: Electromyography
ER-stress: Endoplasmic reticulum stress
FSHD: Fascioscapulohumeral muscular dystrophy
FVC: Forced vital capacity
Gal: Galactose
GGT: Gamma-glutamyl transpeptidase
GlcN: Glucosamine
GNE: N-acetylglucosamine epimerase/ N-acetylmannosamine kinase
HIBM: Hereditary Inclusion Body Myopathy
HPLC: High performance liquid chromatography
iTRAQ: Isobaric tags for relative and absolute quantitation
IVIG: Intravenous immunoglobulin
Man: Mannose
ManN: Oral mannosamine
ManNAc: N-acetylmannosamine
MRI: Magnetic resonance imaging
NCAM: Neural cell adhesion molecule
Neu5Ac: N-Acetylneuraminic acid
NPPV: Non-invasive positive pressure ventilation
PDK1: 3-phosphoinositide-dependent protein kinase 1
PTEN: Phosphatidylinositol 3,4,5-trisphosphate 3-phosphatase and dual-specificity protein phosphatase
QSM: Quadriceps Sparing Myopathy
rAAV: Recombinant adeno-associated virus
ROS: Reactive oxygen species
RT-PCR: Real time polymerase chain reaction
SD: Standard deviation
sIBM: Sporadic inclusion-body myositis
ST-antigen: Thomsen-Friedenreich (T)-antigen sialylated form
UCG: Ultrasound cardiogram
UPR: Unfolded proteins response
Xbp1: X-box-binding protein 1
